# On Singularities and Black Holes in Combination-Driven Models of Technological Innovation Networks

**DOI:** 10.1371/journal.pone.0146180

**Published:** 2016-01-28

**Authors:** Ricard Solé, Daniel R. Amor, Sergi Valverde

**Affiliations:** 1 ICREA-Complex Systems Lab, Universitat Pompeu Fabra, Barcelona, Spain; 2 Institut de Biologia Evolutiva, CSIC-UPF, Barcelona, Spain; 3 Santa Fe Institute, Santa Fe, New Mexico, United States of America; Universidad Veracruzana, MEXICO

## Abstract

It has been suggested that innovations occur mainly by combination: the more inventions accumulate, the higher the probability that new inventions are obtained from previous designs. Additionally, it has been conjectured that the combinatorial nature of innovations naturally leads to a singularity: at some finite time, the number of innovations should diverge. Although these ideas are certainly appealing, no general models have been yet developed to test the conditions under which combinatorial technology should become explosive. Here we present a generalised model of technological evolution that takes into account two major properties: the number of previous technologies needed to create a novel one and how rapidly technology ages. Two different models of combinatorial growth are considered, involving different forms of ageing. When long-range memory is used and thus old inventions are available for novel innovations, singularities can emerge under some conditions with two phases separated by a critical boundary. If the ageing has a characteristic time scale, it is shown that no singularities will be observed. Instead, a “black hole” of old innovations appears and expands in time, making the rate of invention creation slow down into a linear regime.

## Introduction

Technology is one of the most obvious outcomes of human culture. Technological inventions have been developing at an accelerated rate since the industrial revolution [1–5] and economist Brian Arthur conjectured that such rapid growth is a consequence of the underlying dynamics of combination that drives the process [2]. Specifically, it has been suggested that novelties arise mainly as a consequence of new forms of interaction between previous artifacts or inventions [2]. Such view connects the pace of man-made evolutionary designs with a basic principle of biological evolution: the presence of tinkering [6] as a dominant way of generating new structures [7, 8].

Systematic studies of technological change are difficult to perform due to a number of problems. These include the lack of a genome-like description of artifacts and the complex nature of their design paths. The study (largely naturalistic) of some particular systems, such as cornets [9–10] reveals some interesting similarities, while uncovering deep differences with cultural change. More recent work based on network theory [11, 12] provided a novel quantitative approach to technological change that defines a formal framework to explore technological change and the impact of design principles.

One consequence of the combination principle proposed by Arthur is that the growth dynamics of inventions would be faster than exponential (or super-Malthusian) and should exhibit a finite-time singularity [13]. The implications of such rapidly accelerating innovation processes have been discussed in recent years, raising controversial speculations [14]. The superlinear dynamics of innovations has the potential to, e.g., drive cities to unbounded growth, which can easily lead to urban collapse due to limitations in resources [15]. However, our ability to process an abundance of potentially new ideas into usable form may impose significant limits to economic or technological growth [16]. Predicting the progress of technological change is a timely issue but also a difficult task. Nevertheless, some insights have been gathered by dedicated study of available databases and appropriate statistical methods [17, 18].

**Fig 1:**
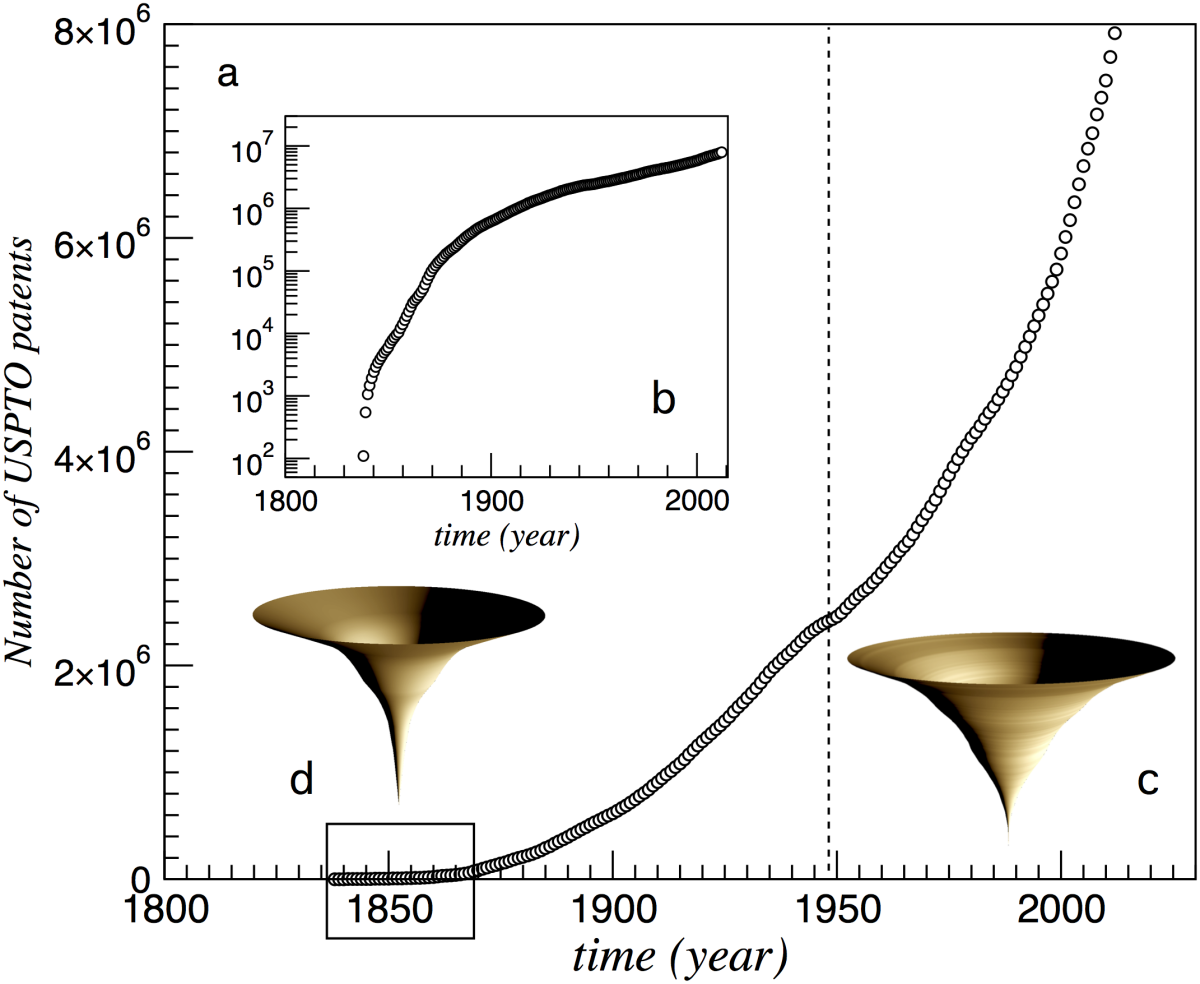
Evolution of technological diversity. The main plot (a) shows the accelerated increase of the total number patents *N*(*t*) as provided by the USPTO dataset. In (b) we show the same data in linear-log form. As an alternative illustration, we also display the spindle diagrams for *N*(*t*) associated to (c) the overall pattern and (d) the early development linked to the Industrial Revolution [corresponding to the period indicated by the open square in (a)].

A surrogate of the ways in which innovations take place in time is provided by patent files [19–21]. Patents are well-defined objects introducing a novel design, method, or solution for a given problem or set of problems. Existing data bases store multiple levels of patent description and they can be analyzed in full detail. Additionally, they indicate what previous novelties have been required to build new ones. An example is given by the U.S. Patent and Trademark Office (USPTO) patents filed between 1835 and 2010 (information about the US filed patents for the mentioned period is available at the official website of the USPTO: http://www.uspto.gov/web/offices/ac/ido/oeip/taf/h_counts.htm). In Fig 1a we display the total number *N* of filed patents, which clearly reveals a superlinear trend over time [3, 13]. The dashed line in particular indicates the start of the modern information technology era (around 1950). We also display the so called *spindle diagrams*, commonly used in paleobiology and archaeology [22] to provide a different visualisation of the diversity expansion process. Here the vertical axis represents time (growing from bottom to top) and the horizontal dimension is associated to diversity: the radius of the circular slice would be proportional to the number of patents filed at that particular time.

In [21] a study of the USPTO database was made in search for evidence of combinatorial evolution. The authors concluded that truly new technological capabilities are slowing down in their rate of appearance, but nevertheless a great deal of combination is present thus allowing for a “practically infinite space of technological configurations” [21]. One potential outcome of this virtually exploding space is a growth dynamics displaying potential singularities, i. e. divergent numbers of inventions would eventually occur as we approach a finite time window. In this paper we want to address the problem of how to define the conditions for technological singularities to be expected. Two main components of combinatorial dynamics will be taken into account: (a) the diversity (number) of potential innovations required to trigger a new one and (b) the degree of ageing that makes older innovations less likely to be used. As shown below, two main phases are expected in this diversity-aging space, defining the conditions for singularities to be present.

## Hyperbolic dynamics: minimal model

Using the simplest approximation, we assume a neutral model of innovation based on pairwise combinations of existing designs. In this model, the set *π* = {*π*_1_, *π*_2_, …, *π*_*N*(*t*)_} defines the “design universe” at any given time *t*. Each *π*_j_ ∈ *π* represents an invention as described, for example, by a patent file. Here, *N*(*t*) is the total number of inventions (patents) at year *t*. Two given designs will then combine at a given time *t* with a given probability: $\pi_{{}_{i}}+\pi_{{}_{j}}\overset{\mu_{ij}}{\to }\pi_{{}_{N(t)+1}}$ where *μ*_*ij*_ weights the likelihood of such an event to happen. This defines a second-order (bimolecular) reaction kinetics [23–25]. Such nonlinear reaction dynamics seems to pervade the super-exponential growth observed in a number of economic and demographic systems [26–29].

How is this space expanded? We will assume that every element *π_j_* ∈ *π* has the same potential to attach to other existing elements. We can consider a more complex kinetic equation, namely:

$$\begin{array}{c} \frac{dN}{dt}=\mu \left(t\right)N^{1+z} \end{array}$$ (1)

where *μ*(*t*) is the attachment rate of inventions at a given time. The parameter 0 ≤ *z* ≤ 1 weights the departure from the linear scenario. For *z* = 0 the above yields to exponential growth. If a pure (pairwise) combination scenario were at work, we would see *z* = 1, since any pair of inventions is likely to interact. By solving eq (1) we obtain

$$\begin{array}{c} N\left(t\right)={\left[N^{-z}\left(0\right)-z\int_{0}^{t}\mu \left(\tau \right)d\tau \right]}^{-\frac{1}{z}} \end{array}$$ (2)

For the simplest scenario where *μ* can be considered constant, i. e. $\mu =\langle \mu \left(t\right)\rangle =\left(\int_{0}^{t}\mu \left(\tau \right)d\tau \right)/t$, we can write the previous equation as follows:

$$\begin{array}{c} N\left(t\right)={\left(z\mu \right)}^{-1/z}{\left(t_{s}-t\right)}^{-1/z} \end{array}$$ (3)

where *t*_*s*_ is a finite time given by $t_{s}=1/\left(z\mu N_{0}^{z}\right)$ with *N*_0_ = *N*(0). A very interesting feature of this solution is the presence of a *singularity*: as we approach *t*_*s*_, a divergence occurs in *N*. For the pure combination solution with *z* = 1 we would observe a growth curve following:

$$\begin{array}{c} N\left(t\right)=\frac{1}{\mu }\left(\frac{1}{t_{s}-t}\right) \end{array}$$ (4)

which provides a prediction of how invention numbers will increase under a neutral model where all inventions (patents) are equally likely to combine.

The results presented in [21] suggest that an exponential phase is followed by another phase that deviates from the hyperbolic growth picture when time is used as the horizontal axis. However, the *m*-order recombination of existing technologies has the potential for accelerating the appearance of innovations without bounds (see, Fig 2 in [21]). In this paper we aim to develop a model of technological evolution that takes into account the combinatorial nature of the process and potential mechanisms of ageing that can slow down the hyperbolic dynamics. The model does not intend to reproduce the time series provided by the patent data set. Instead, it incorporates a minimal set of assumptions associated to an abstract set of interacting innovations beyond the pairwise, second-order reaction metaphor.

## Generalized models

Several simplifications have been made in the model above. One is that a limited number of previous innovations are combined to obtain a new one. Another is that we assumed by default that all innovations can (in principle) contribute to future technologies, when actually many will become obsolete. Some type of ageing factor needs to be considered. Such ageing has been found to be present in different types of growing networks [30–32] including different forms of collaboration among scientists and links among innovations [20] and will be also studied here. In this paper we do not consider an explicit network architecture. Instead, we define a so called mean-field model where links among innovations are implicit.

One way of including multiple innovations is to consider the average number *k* of inventions that are used to obtain new ones. On the other hand, we need to define the way two elements might interact. This can be done by considering a generalised integral equation:

$$\frac{dN}{dt}=\int_{0}^{N}\underset{⃛}{k}\;\int_{0}^{N}\Gamma (\tau_{1},\ldots ,\tau_{k})d\tau_{1}\ldots d\tau_{k}$$ (5)

Here the kernel Γ(*τ*_1_, …, *τ*_*k*_) defines the probability that *k* different patents interact in order to give a new invention. Note that we have considered the origin as our lower integration bound, since (according to our continuous notation) any positive real value of *τ*_*i*_ could contribute to recombination. This general expression contains the hyperbolic scenario introduced above as one special case when all elements can equally interact and thus Γ = *μ*. To see this, notice that we have now

$$\begin{array}{c} \frac{dN}{dt}=\int_{0}^{N}\int_{0}^{N}\mu d\tau_{1}d\tau_{2}\\ =\mu \left(\int_{0}^{N}d\tau_{1}\right)\left(\int_{0}^{N}d\tau_{2}\right)=\mu N^{2} \end{array}$$ (6)

From now on we will assume that the kernel can be factorized: all inventions can interact in similar ways and thus

$$\begin{array}{c} \Gamma \left(\tau_{1},...,\tau_{k}\right)=\prod_{l=1}^{k}\Gamma \left(\tau_{l}\right). \end{array}$$ (7)

In that case, the previous eq (5) reads now:

$$\frac{dN}{dt}=\int_{0}^{N}\;\underset{⃛}{k}\;\int_{0}^{N}\prod_{l=1}^{k}\Gamma (\tau_{l})d\tau_{1}\ldots d\tau_{k}$$ (8)

and thus our general model to be explored below reads now:

$$\begin{array}{c} \frac{dN}{dt}=\prod_{l=1}^{k}\left[\int_{0}^{N}\Gamma \left(\tau_{l}\right)d\tau_{l}\right] \end{array}$$ (9)

### Power law aging

The integral eq (10) contains the number of innovations required to further expand the technological space. Now we need to introduce how ageing affects the range of interactions. One choice is a power law kernel, namely

$$\begin{array}{c} \Gamma \left(\tau_{l}\right)∼\mu^{1/k}{\left(N-\tau_{l}\right)}^{-\gamma } \end{array}$$ (10)

where the scaling exponent *γ* ≥ 0 gives a measure of how fast previous innovations become obsolete and are not incorporated. This kernel has been used in different contexts, including in the analysis of collaborations among researchers, which is a closely related problem [18]. In this case, the general model is written as

$$\begin{array}{c} \frac{dN}{dt}=\prod_{l=1}^{k}\left[\int_{0}^{N}\mu^{1/k}{\left(N-\tau_{l}\right)}^{-\gamma }d\tau_{l}\right] \end{array}$$ (11)

If we assume equivalence between all the components of our system, all kernels being equal we obtain here:

$$\begin{array}{c} \frac{dN}{dt}={\left[\int_{0}^{N(t)}\mu^{1/k}{\left(N-\tau \right)}^{-\gamma }d\tau \right]}^{k}\\ =\frac{\mu }{{\left(1-\gamma \right)}^{k}}N^{(1-\gamma )k} \end{array}$$ (12)

By solving this equation, we can show that the solution reads:

$$\begin{array}{c} N\left(t\right)={\left[C_{0}+\eta \left(k,n\right)t\right]}^{1/(1-(1-\gamma )k)} \end{array}$$ (13)

The constants are defined by $C_{0}=N_{0}^{1-(1-\gamma )k}$ and

$$\begin{array}{c} \eta \left(k,n\right)=\frac{1-(1-\gamma )k}{{\left(1-\gamma \right)}^{k}} \end{array}$$ (14)

respectively. This equation will be consistent with a singularity provided that the scaling exponent is negative. This leads to a critical condition:

$$\begin{array}{c} k>k_{c}=\frac{1}{1-\gamma } \end{array}$$ (15)

**Fig 2:**
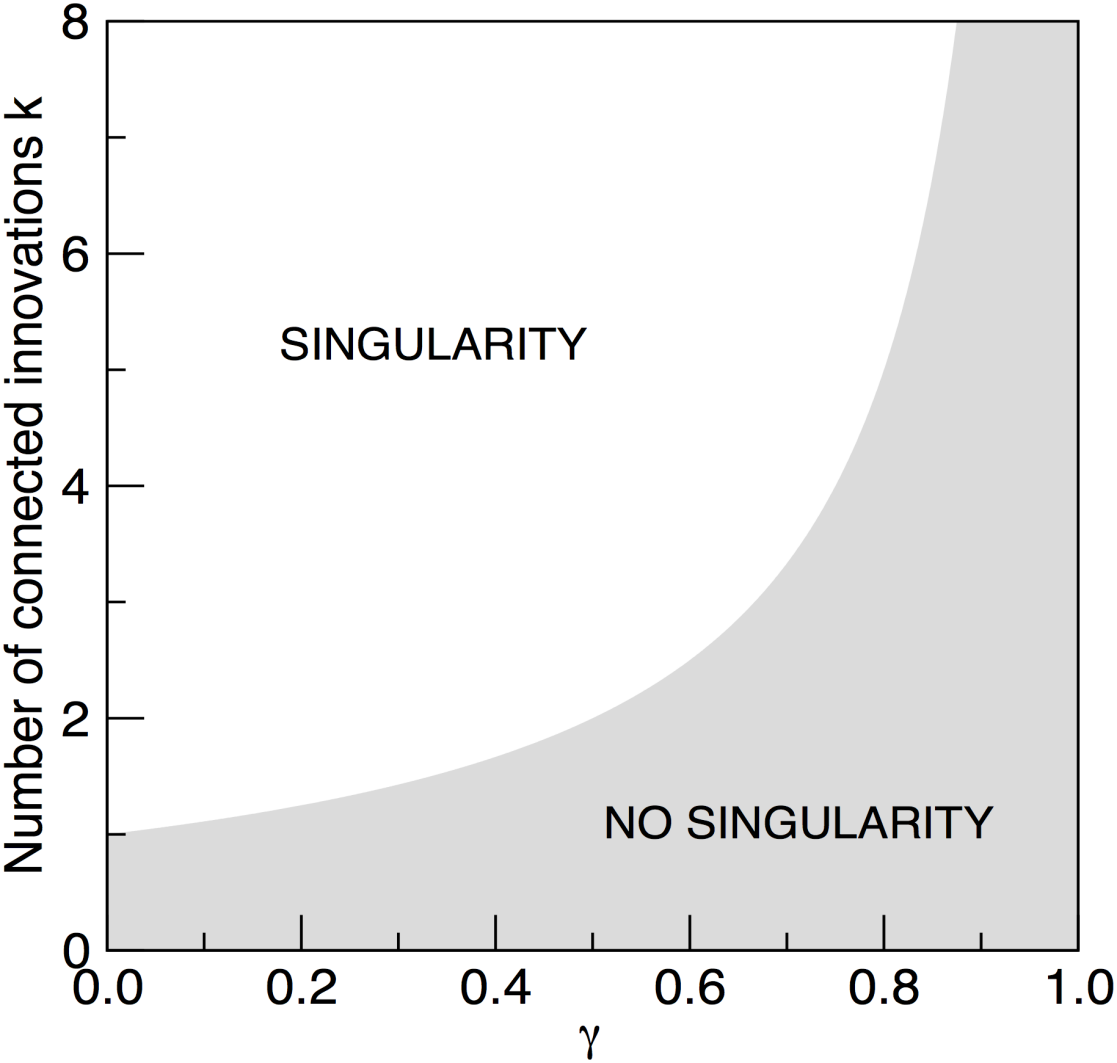
Two phases predicted by the generalised model of technological evolution with power law ageing. The white area includes all parameter combinations allowing a singularity to emerge through a hyperbolic growth process.

The phase diagram predicted by this critical boundary is shown in Fig 2, where we plot *k*_*c*_(*γ*). The two domains showing or lacking a singularity are separated by this curve. As we can see, singularities are expected even for *γ* = 0 provided that *k* > 1. Similarly, when *k* = 2 we have the standard pairwise reaction scheme described above.

### Exponential ageing (*k* = 2)

The power law kernel introduces a long tail and thus long-memory effects. Although links with old inventions are much rare, they can be established and thus a contribution will always be expected. What is the impact of using a different type of interaction kernel involving a more rapid decay that forbids new inventions to “connect” with very old ones? This can be modelled with an exponential decay of the form *e*^−*γ*(*N*−*α*)^. As defined, the smaller the value of *γ*, the longer the age (namely, *N*−*α*) that patents can reach while being still able to generate new inventions. Indeed, in the limit *γ* → 0 all the inventions would equally contribute (no matter their age), recovering again the simple hyperbolic (pairwise) scenario analyzed in section 2.

**Fig 3:**
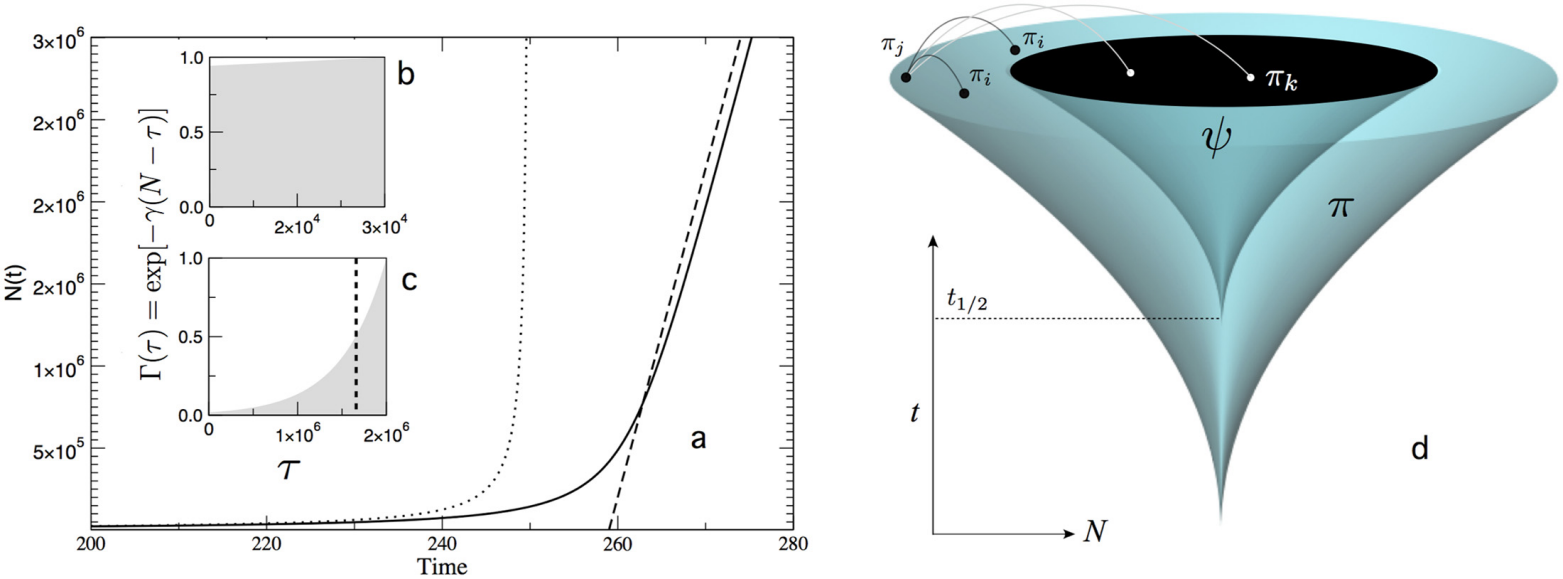
Transient hyperbolic growth and blackholes in combination models with limited memory. In (a) we display our predicted growth curve *N*(*t*) and two approximations considering short time (dotted line) and long term (dashed line) scales. The effective kernels for these two scales are displayed in the inset plots (b) and (c). The maximum value displayed in the *τ* axis of insets (b) and (c) corresponds to *N*(*t*) at times *t* = 213 and *t* = 270, respectively. The characteristic scale of ageing imposed by the kernel implies that there is a time horizon beyond which no connections among inventions can be made. This is illustrated schematically in (d) where we show the spindle diagram of the whole system *π* along with a subset *ψ*(*t*), first appearing at the characteristic time *t*_1/2_ where the probability of citing the oldest invention *π*_1_ is half the maximum. All inventions within this “black hole” will be disconnected from the rest. In the present (top large circle) only new inventions (filled circles) occupying the outer part of the circle can connect among them whereas they cannot link (light lines) with those in the black hole (open circles). The parameter values used in (a)-(c) correspond to *μ*′ = 2 × 10^5^, *t*_0_ = 0, *N*_0_ = 5000, and *γ* = 2 × 10^−6^.

In order to illustrate the impact of this limited memory, let us consider again the pairwise (*k* = 2) scenario. The generation of new patents is now given by

$$\begin{array}{c} \frac{dN}{dt}=\mu \prod_{l=1}^{2}\left[\int_{0}^{N}e^{-\gamma (N-\tau_{l})}d\tau_{l}\right]\\ =\mu^{\prime }{\left[e^{-\gamma N}-1\right]}^{2} \end{array}$$ (16)

where *μ*′ = *μ*/*γ*^2^. By solving this equation we obtain an implicit form:

$$\begin{array}{c} \left(N-N_{0}\right)+\frac{1}{\gamma g_{\gamma }\left(N\right)}-\frac{1}{\gamma g_{\gamma }\left(N_{0}\right)}+\\ +\frac{1}{\gamma }\ln\left[\frac{g_{\gamma }\left(N\right)}{g_{\gamma }\left(N_{0}\right)}\right]=\mu^{\prime }\left(t-t_{0}\right). \end{array}$$ (17)

where we used the notation *g*_*γ*_(*x*) ≡ *e*^−*γx*^ − 1. This equation can be numerically solved and the result shown in Fig 3a for a given set of parameters. We can appreciate from this diagram that there is a delayed growth phase at the beginning followed by an apparently linear growth in late stages. In other words, the dynamics has no singularity. Is that the case? Although solving the general problem can be extremely cumbersome, we can deal with some approximations that can be applied at different stages of the system.

The first considers the case *γ* < < 1 and the initial phase of the expansion. If *N* is not large then *γN* < < 1 and we can use the approximation *e*^−*γN*^ ≃ 1 − *γN* on eq (17), leading to:

$$\begin{array}{c} N-N_{0}-\frac{1}{\gamma^{2}}\left[\frac{1}{N}-\frac{1}{N_{0}}\right]+\\ +\frac{1}{\gamma }\ln\left[\frac{N}{N_{0}}\right]=\mu^{\prime }\left(t-t_{0}\right). \end{array}$$ (18)

Given the condition *γ* < < 1, the LHS of the previous equation is governed by the quadratic terms of *γ*. Hence, neglecting the first and last terms on the LHS, we obtain the following approximate solution for the evolution of *N* at initial stages of technological evolution:

$$\begin{array}{c} N\left(t\right)=\frac{1}{\gamma^{2}\mu^{\prime }\left(t_{s}-t\right)} \end{array}$$ (19)

where *t*_*s*_ = 1/(*μ*′ *γ*^2^ *N*_0_) + *t*_0_. The previous equation predicts a singularity at some time in the future although such singularity is in conflict with our approximation and the hyperbolic growth is only a transient phenomenon.

Let us now focus on the long-term (*t* → ∞) dynamics of the system. In this case it is sensible to assume a very large number of existing patents, and hence *e*^−*γN*^ → 0. In this case, we can rewrite eq (17) as:

$$\begin{array}{c} \left(N-N_{0}\right)-\frac{1}{\gamma }+\frac{1}{\gamma^{2}N_{0}}-\frac{1}{\gamma }\ln\left[\gamma N_{0}\right]=\mu^{\prime }\left(t-t_{0}\right), \end{array}$$ (20)

where we have used $e^{-\gamma N_{0}}≃1-\gamma N_{0}$. Thus, from eq (20) it is straightforward to obtain the long-term solution for the dynamics of *N*:

$$\begin{array}{c} N\left(t\right)=\mu^{\prime }\left(t-t_{0}\right)+\varepsilon \end{array}$$ (21)

with *ε* = *N*_0_ + 1/*γ*−1/(*γ*^2^ *N*_0_) + ln[*γN*_0_]/*γ*.

Eq (21) reveals that *N* exhibits a linear growth dynamics when large values of *t* are considered. Note that this long-term dynamics is notably different from the hyperbolic dynamics predicted for initial stages of evolution (see the explanation above). In Fig 3 we show the agreement between these approximations and the exact solution (obtained numerically). As we can see, the analytic results confirm that the initial hyperbolic trend (dotted curve) is eventually replaced by a slowdown characterised by a linear process (dashed line) with no technological singularity associated.

An intuitive explanation for the change from early hyperbolic to late linear dynamics is provided in the insets of Fig 3b and 3c. Here we show the kernel associated to early (Fig 3b) and late (Fig 3c) times, and thus smaller and larger numbers of innovations. Although the area covered by Γ almost fills the plot when *N* ∼ *O*(10^4^), it becomes smaller with large *N* values (here *N* ∼ *O*(10^6^)). In Fig 3b, the probability of recombining any existing patent is higher than 90% of the maximum [i.e., 90% of the probability of recombining the newest pattent *N*(*t*), with *t* = 213]. However, the probability of combining patents existing at *t* = 270 (Fig 3c) folds to approximately zero for the oldest patents. Then, we can arbitrarily define a patent number *π*_*h*_(*t*) that delimits the frontier between up-to-date patents and obsolete patents (which will hardly ever been recombined again). Specifically, we consider *π*_*h*_(*t*) to be the patent number for which the probability of recombination is half the maximum (as indicated by the dashed line in Fig 3c). Thus, the effect of ageing (or loss of memory) dominates in the long term (Fig 3a and 3c) and the effective rate of innovation become linear. Such slowdown prevents the system from approaching a divergent dynamics.

These results can be graphically interpreted as shown in Fig 3d. Here we use again the spindle diagram showing how the universe (or space) of innovations experiences an accelerated growth at early stages of development. Novel patents such as *π*_*j*_ will be distributed over the outer parts of the patent space (a circle at each time step) and connect with others such as *π*_*i*_. After a critical time *t*_1/2_, some inventions start to become obsolete or forgotten. From this time on, the expansion speed stabilises, and both the universe of inventions and the “black hole” *ψ*(*t*) at its center (which represents the area of obsolete technology) grow at the same constant speed. Inventions within the black hole (such as *π*_*k*_ in Fig 3d) cannot be used and thus no information about them can cross the obsolescence frontier. Our technological memory establishes the distance between these two boundaries in the innovations space, and this distance determines the number of up-to-date inventions, which in turn determines the expansion rate of the innovation space.

### Exponential ageing (*k* ≥ 1)

The previous results can be generalised to the *k*-diversity scenario, where the new equation reads

$$\begin{array}{c} \frac{dN}{dt}=\mu \prod_{l=1}^{k}\left[\int_{0}^{N}e^{-\gamma (N-\tau_{1})}d\tau_{1}\right] \end{array}$$ (22)

This general model leads to:

$$\begin{array}{c} \int_{N_{0}}^{N}\frac{d\tilde{N}}{{\left[e^{-\gamma \tilde{N}}-1\right]}^{k}}=\mu^{\prime }\int_{t_{0}}^{t}d\tilde{t} \end{array}$$ (23)

which, at initial stages reads:

$$\begin{array}{c} \int_{N_{0}}^{N}\frac{d\tilde{N}}{{\left(\gamma \tilde{N}\right)}^{k}}=\mu^{\prime }\int_{t_{0}}^{t}d\tilde{t}, \end{array}$$ (24)

and gives hyperbolic dynamics, whereas in the long-term dynamics we have now:

$$\begin{array}{c} \int_{N_{0}}^{N}d\tilde{N}=\mu^{\prime }\int_{t_{0}}^{t}d\tilde{t}, \end{array}$$ (25)

again leading to linear dynamics.

### Aging in a multiple order recombination model

The previous models provide a simple theoretical framework to study the dynamics of purely combinatorial innovations following a *k*-order kinetics. However, it seems sensible to think that recombination takes place involving different *k*’s. In particular, we should consider one special case where both combination and novel innovations contribute to the growth of the technological universe. What would be the impact of considering *k* = 1 as part of the dynamical equations? Here we present a simple, revealing example that deals with a bimodal recombination process.

In previous sections we have described the pairwise recombination in which two different innovations are involved. The corresponding unimodal recombination equation is given by Eq (23) using *k* = 2. Let us here consider that an additional mode of recombination is present in the system. A very simple model that can be considered is the first-order recombination *k* = 1 in Eq (23). This kind of recombination can be seen as a mutation of an existent patent (e.g., a given patent is for the first time applied in a new field, thus giving rise to a new patent). In the absence of ageing effects, it is easy to see that *k* = 2 leads to hyperbolic growth and, analogously, *k* = 1 would lead to Malthusian (exponential) growth dynamics. Considering a bimodal recombination dynamics in which the two previous modes are added up, we have:

$$\begin{array}{c} \frac{dN}{dt}=\mu_{M}\int_{0}^{N}e^{-\gamma_{M}\left(N-\tau_{1}\right)}d\tau_{1}+\mu_{H}\int_{0}^{N}e^{-\gamma_{H}\left(N-\tau_{1}\right)}d\tau_{1}\int_{0}^{N}e^{-\gamma_{H}\left(N-\tau_{2}\right)}d\tau_{2} \end{array}$$ (26)

where *μ*_*M*_ and *γ*_*M*_ stand for the attachment rate and aging exponent for the mode *k* = 1, respectively, while *μ*_*H*_ and *γ*_*H*_ are the analogous parameters for the hyperbolic mode *k* = 2.

**Fig 4:**
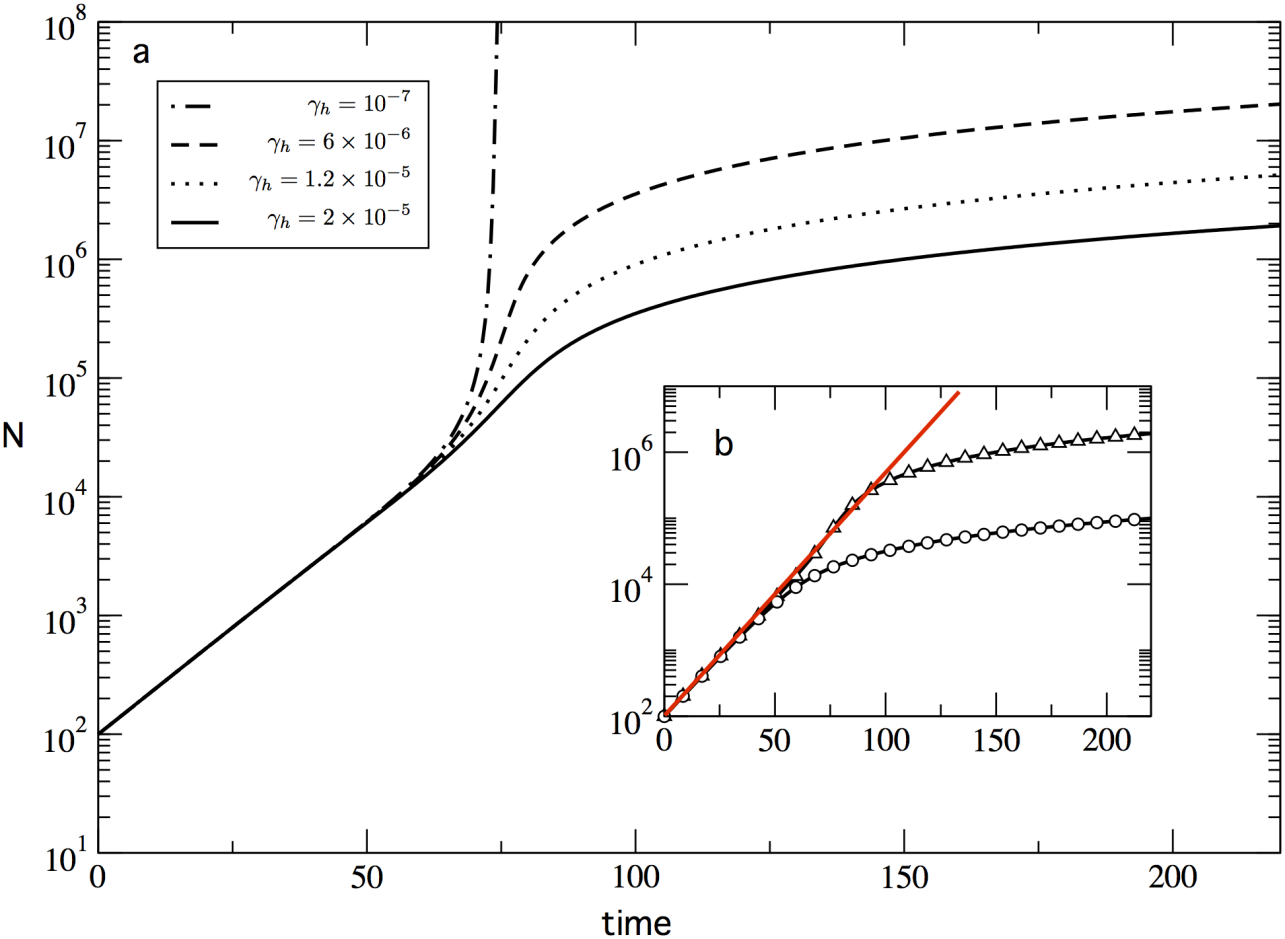
Innovation dynamics of the bimodal recombination model. In (a) we show the different dynamics of *N*(*t*) for different aging decays affecting the hyperbolic (*k* = 2) term. The corresponding *γ*_*H*_ values are indicated in the legend. The rest of the parameter values are: *μ*_*M*_ = 8.5 × 10^−2^, *γ*_*M*_ = 1.5 × 10^−4^ and *μ*_*H*_ = 5 × 10^−7^. In (b) we show again the case *γ*_*H*_ = 2 × 10^−5^ (triangles), and the accumulated contribution of the Malthusian (*k* = 1) recombination to the total patent number *N*. At any time, the accumulated number of patents that have been generated by hyperbolic (*k* = 2) recombination corresponds to the difference between the two curves. The red line corresponds to an exponential fit to the first part (up to *t* = 50) of the time series.

In contrast with the unimodal cases above, finding the analytical solution of the bimodal equation (27) can be cumbersome. Fig 4 shows the numerical solution of equation (27) for different values of the aging parameter *γ*_*H*_. As shown in the previous sections, the hyperbolic growth term *μ*_*H*_ is responsible of a superexponential [higher than linear in the semi-logaritmic axis displayed by Fig 4] growth in the number of innovations. However, this accelerated trend can be counterbalanced by the aging effects. This is illustrated in Fig 4a, for which we observe that the system shifts from an approximately divergent dynamics when the ageing parameter *γ*_*H*_ is relatively low (see the dashed-dotted line), to a linear dynamics for high values of *γ*_*H*_ (dashed, dotted and solid lines). Moreover, we observe that the accelerated trend observed slightly before *t* = 75 decays as the ageing effects increase. In Fig 4b we show the accumulated contribution that the first-order (*k* = 1) recombination makes to the system. We can see that early exponential growth of *N* (*t* < 50) can be mainly attributed to this first-order recombination. After a given number of patents is accumulated (around *t* = 50), the second-order recombination becomes important, and *N* departs from the first-order regime. Interestingly, Fig 4b reminds the behaviour observed in the number of patents (see Fig 1 in Ref. [21]), specially if our first-order term is identified as the production of new kinds of technology (that could be related to the number of UPSTO codes), and the hyperbolic recombination as the emergence of new patents based on two existing technologies. This is of course a first approximation to the actual role played by codes versus patents, but it illustrates a potential mean field approach to these observations.

## Discussion

The nature and tempo of innovation is a difficult and timely topic. It has been the focus of attention from evolutionary biologists, economists and physicists alike. Inventors get inspiration from previous, existing designs, while they push forward the boundaries of invention. In searching for a theory of technological change, the combinatorial nature of technology seems to be an essential component of human creativity. By combining previous designs into novel ones, there is a potential for an explosion of novelties, which could eventually move towards a singularity. How can we test such possibility? Patent files are a privileged window into such process, since they provide a first approximation to both the growth of inventions and their interactions over time. The accelerated pattern of patent growth suggests that a super linear process of innovation is taking place and available evidence indicates that this is at least partially associated to combinatorial processes [21].

In this paper we have explored a simple class of models that include both the richness of combinations and how rapidly the relevance of previous inventions fades with time. These two features can be seen as two opposing forces: the diversity of potential previous inventions to be combined powers combinatorial design, while the obsolescence of the same inventions makes them less likely to contribute to combinations. Our goal was not as much as to fit data than understand the basic scenarios where singularities might emerge when both features are included.

We have shown that long-memory kernels permit the presence of singularities under some conditions, while kernels involving a characteristic time scale of ageing forbid divergences to occur. The first class predicts two different phases, which reminds us of a picture of innovation defining a phase transition between sub-critical and super-critical phases [33]. The second provides a plausible reason why singularities might fail to be observed, while the transient dynamics of innovation appears hyperbolic. Further investigations should analyse other temporal trends (including the patterns of fluctuations) associated to these class of models and a more detailed analysis of available time series. Existing models of evolution of innovations [34, 35] can provide very useful tests to the ideas outlined here. Other factors have not been considered here, such as the limited resources effectively available for developing new technologies. Nevertheless, our models suggests that some generic trends can be defined that pervade the ways in which innovation evolves.
